# When the Frequencies of Sensitization and Elicitation of Allergic Reaction Do Not Correlate—The Case of Apple Gibberellin-Regulated Protein Tested in an Italian Population

**DOI:** 10.3389/falgy.2021.745825

**Published:** 2021-10-21

**Authors:** Lisa Tuppo, Claudia Alessandri, Ivana Giangrieco, Maurizio Tamburrini, Ricardo Hernandez Arriaza, Maksymilian Chruszcz, Adriano Mari, Maria Antonietta Ciardiello

**Affiliations:** ^1^Institute of Biosciences and BioResources (IBBR), CNR, Naples, Italy; ^2^Allergy Data Laboratories (ADL) S.r.l., Latina, Italy; ^3^Associated Centers for Molecular Allergology (CAAM), Rome, Italy; ^4^Department of Chemistry and Biochemistry, University of South Carolina, Columbia, SC, United States

**Keywords:** apple GRP, applemaclein, food allergy, Pru p 7, IgE-binding, sensitization frequency, applemaclein molecular model

## Abstract

**Background:** The literature reports describing allergic symptoms against apples in the patients sensitized to the gibberellin-regulated proteins (GRPs) suggested the presence of an allergenic GRP in this fruit.

**Objective:** This study aimed to assess the presence of a GRP protein in apples and investigate its allergenicity.

**Methods:** The protein was isolated and identified by the classical biochemical methods. The bioinformatics tools were used for similar searches and molecular modeling. The immunological features were investigated using the multiplex FABER test. Clinical data were collected by the allergy specialists.

**Results:** A GRP was detected in the apple peel and pulp and it was named applemaclein. This protein displays 94% of sequence identity with peamaclein, Pru p 7, representing the prototype of this allergen family. The applemaclein molecular model shows a very irregular surface with grooves/clefts that may potentially accommodate small molecular ligands. In a population of 4,721 patients in Italy, 187 (4.0%) were sensitized to any allergenic GPR. Of those, 115 (61.5%), 61 (32.6%), 30 (16.0%), and 99 (52.9%) had immunoglobulin E (IgE) to apple, peach, pomegranate, and cypress GRP, respectively. However, in a cohort of the patients in Italy, most individuals IgE positive to the apple GRP did not report allergic reactions against this fruit.

**Conclusion:** Compared with the peach Pru p 7, applemaclein shows some different structural features and higher sensitization frequency, which is often not associated with allergic reactions against apple. Further studies are needed to understand a possible correlation between the applemaclein structural properties, the interaction with still unknown molecules, and immunological behavior.

## Introduction

The members of the gibberellin-regulated protein (GRP) family have been identified as allergens quite recently and they are currently attracting much attention. The GRPs are basic, small proteins (7 kDa) stabilized by six disulphide bridges. Similar to other allergens, such as non-specific lipid trasfer proteins (nsLTPs), they are stable for heating and gastrointestinal digestion (1). Peamaclein, the peach GRP, is the first member of this protein family to be identified and registered as an allergen (Pru p 7). Its detection was driven by the clinical history of a patient reporting anaphylaxis after peach consumption, but the patient had proved to be immunoglobulin E (IgE) negative to the isolated peach allergens available at that time (2). Then, Pru p 7 was associated with severe allergy symptoms and proposed as a marker allergen related to systemic reactions in peach allergy (3–5). In the meanwhile, the allergenic homologous proteins were identified in other fruits, such as Pru m 7 in Japanese apricot (4), Pun g 7 in pomegranate (6), Pru av 7 in cherry (WHO/IUIS), and Cit s 7 in orange (7). More recently, an allergenic GRP was identified in the pollen of *Cupressus sempervirens* (Cup s 7), and its IgE-binding competition with Pru p 7 and Pun g 7 was demonstrated (8). Cross-reaction between Cup s 7 and the homologous proteins from *Cryptomeria japonica* (Cry j 7) and *Juniperus ashei* (Jun a 7) pollens was also observed (9). In addition, a frequent association between the GRP-related food allergy and *Cupressaceae* pollen sensitization, especially occurring in the areas with high exposure to this type of tree pollen, was reported (9, 10). The cross-reactivity between the GRPs was suggested as a cause of multiple fruit allergies and the clinical cross-reactivity, at least among peach, Japanese apricot, orange, and pomegranate was proven (11). In addition to these fruits, the patients sensitized to GRP are frequently reported to experience allergic reactions against other fruits, such as apples (4, 12).

Apple is one of the most consumed fruits in the world. Nevertheless, in some subjects, it can cause different patterns of allergic reactions, from oral allergy syndrome (OAS) to severe systemic reactions. Sometimes, allergy to apple is associated with pollinosis, especially in the patients sensitized to Bet v 1 (13). Four allergens, Mal d 1–4, have been so far identified in this fruit and registered by the WHO/IUIS. Mal d 1 is a Bet v 1-like protein, Mal d 2 a thaumatin-like protein, Mal d 3 a 9 kDa nsLTP, and Mal d 4 a profilin (14, 15). The published observation that the patients sensitized to GRP frequently reported allergic symptoms against apple (4, 12), which, such as peach, taxonomically belongs to the *Rosaceae* family, suggested the presence in this fruit of a still uncharacterized allergen belonging to the GRP family.

In this context, this study aimed to investigate the presence of GRP in the peel and pulp of the apple fruit, the isolation of the protein, and the analysis of its structural and immunological properties. The specific objects of this study included the analysis of (i) the structural features by similarity search and molecular modeling, (ii) the frequency of sensitization to the apple GRP in a random population of suspected allergics, (iii) the frequency of sensitization in a subpopulation of the patients sensitized to GRP, (iv) reported allergy symptoms of patients following the apple ingestion and (v) a comparative analysis of the structural and immunological features of apple GRP with homologous allergenic proteins.

## Materials and Methods

### Purification of Applemaclein From Apple Peel Extract

Apple fruits (*Malus domestica*), cultivar Annurca and Golden Delicious, were purchased in a local market (Naples, Italy). The peel and pulp were manually separated and used to investigate the presence of GRP. Next, the peel of Annurca cultivar was chosen as starting material to purify the GRP analyzed in this study, following the already reported procedure (6), with a few modifications. Briefly, the fruit sample was homogenized in a blender with 10 mM Tris-HCl, pH 8.0 (1:0.7 w/v). After 2 h at 4°C, the homogenate was centrifuged at 17,300 x *g*. The supernatant, representing the protein extract, was collected, dialyzed against 10 mM Tris-HCl, pH 7.2 (buffer A), and loaded on a DE52 (Whatman, Brentford, UK) column, equilibrated in the same buffer. An apple GRP was eluted in the column flow-through, and then loaded on an SP-Sepharose column (Amersham Biosciences, Uppsala, Sweden), equilibrated in buffer A. Elution was carried out by increasing the NaCl concentration. Further purification was performed on a HiLoad 16/600 Superdex 75 prep grade column (GE Healthcare Milan, Italy) using an ÄKTA pure protein purification system (GE Healthcare), equilibrated in 10 mM phosphate buffer, pH 7.4, containing 0.25 M NaCl. Afterward, the fractions containing the protein purified to homogeneity were pooled, concentrated, and desalted by ultrafiltration on the Ultracel 3K Amicon Ultra filters (Millipore, Carrigtwohill, Ireland).

During the purification procedure, the apple GRP was followed by RP-HPLC. The protein concentration was estimated on the basis of the molar extinction coefficient at 280 nm (6,710 M^−1^ cm^−1^) calculated using the protein sequence and the ProtParam tool on the Expasy platform (16).

### Analysis by SDS-Page

The extract and the purified protein were analyzed by reducing 15% Sodium Dodecyl Sulphate - PolyAcrylamide Gel Electrophoresis (SDS-PAGE) on a Bio-Rad Mini Protean apparatus (Biorad, Segrate, Italy).

### Analysis by RP-HPLC

The peel extract and the purified protein were analyzed by Reversed Phase-High Performance Liquid Chromatography (RP-HPLC) on a Vydac (Deerfield, IL, USA) C8 column (4.6 mm x 250 mm), using a Beckman System Gold apparatus (Fullerton, CA, USA). Elution was performed by a multistep linear gradient of eluent B (0.08% TFA in acetonitrile) in eluent A (0.1% TFA) at a flow rate of 1 ml/min. The eluate was monitored at 220 and 280 nm. The separated fractions were manually collected and analyzed.

### Amino Acid Sequencing

About 400 pmol of the purified protein were subjected to automated direct amino acid sequencing of the N-terminal region with a Protein Sequencer PPSQ−33B (Shimadzu Corporation, Tokyo, Japan).

### Stability to the Simulated Gastric and Intestinal Digestion

*In vitro* digestions by simulated gastric fluid (SGF) and simulated intestinal fluid (SIF) were performed as reported for Pru p 7 in Tuppo et al. (1). Then, 30 μg of native applemaclein were subjected to digestion and then analyzed by RP-HPLC and SDS-PAGE. The details are reported in the Supplementary Material.

### Specific IgE Detection With the Faber^®^ Multiplex Testing System

The FABER^®^ test (ADL S.r.l., Latina, Italy) is a multiplex *in vitro* serological test allowing the detection of specific IgE antibodies from allergic subjects (14, 17). This test was adopted to analyze sera of patients suffering from suspected allergies. The data were obtained from a set of biochips including, in addition to Pru p 7, Pun g 7, and Cup s 7, also applemaclein, spotted for experimental purposes. Before the immobilization on the FABER biochip, applemaclein was coupled to nanobeads following the same procedure applied to all the other allergenic preparations. The FABER test allowed the detection of specific IgE to each of the allergenic preparations contained in the regular FABER biochip, also including apple GRP in single testing.

### IgE Binding Inhibition

Immunoglobulin E inhibition experiments were performed using the Single Point Highest Inhibition Achievable assay (SPHIAa) (8, 18) with some modifications. Briefly, 0.12 ml of a single undiluted serum was co-incubated with 0.12 ml of a solution containing 0.1 mg of the purified protein. Next, the IgE binding inhibition was evaluated by running the FABER test and recording the residual IgE binding on the allergens spotted on the biochip. The reference values for the lack of IgE binding inhibition were obtained for each serum by running the control samples where the allergen solution was replaced with buffer only. The inhibition values were calculated in real time by a specific procedure developed within the InterAll software (version 5.0, ADL, Latina, Italy).

### Patients

The clinical data of patients and allergy diagnostic results were recorded by an allergy specialist and transferred in real time from the laboratory into the InterAll software (version 5.0, ADL, Latina, Italy), a customized allergy electronic record for diagnostic and clinical data storing. The specific clinical information was collected using the standard questionnaire reported in a previous study (19). Data from 4,721 subjects consecutively tested for suspected allergy on the FABER biochip were analyzed and a subset of 187 patients IgE positive to at least one GRP was detected and further investigated. The reliable clinical data were available only for 1,000 patients which asked for a medical examination directly to the allergologists of the Associated Centers for Molecular Allergology (CAAM), Rome, Italy.

To perform the SPHIAa assay, five characterized sera (numbered 1–5) were selected from the InterAll data bank (version 5.0, ADL) because they had been shown to contain IgE specifically binding the peach peamaclein, Pru p 7, following the FABER test (Supplemenatary Table 2). Four of them (1–4) proved to be IgE positive to the pomegranate homologous protein, Pun g 7.

### Computational Analysis of GRPs Sequences and Three-Dimensional Structures

The three-dimensional models of applemaclein, as well as Cit s 7.0101, Cry j 7.0101, Cup s 7.0102, Jun a 7.0101, Pru av 7.0101, Pru m 7.0101, Pru p 7.0101, and Pun g 7.0101 were generated using a Swiss Model (20) and crystal structure of potato Snakin-1 (20) as a template. The models were visualized, compared, and analyzed using COOT (21) and PyMOL (22). Adaptive Poissons-Boltzmann Solver (APBS) (22) as implemented in PyMOL was used to calculate the distribution of charges on the applemaclein surface. DALI (23) and PDBeFOLD (24) servers were used to search for structural homologs. PROFUNC (25) was used to search for DNA and ligand binding motifs. Sequence alignment was prepared with MUSCLE (26). Jalview (20) and PyMOL were used to prepare the figures.

## Results

### Purification of Apple GRP From the Fruit Peel

The protein extract from apple peel was prepared as described in Materials and methods Section. The extract protein profile obtained by RP-HPLC displayed several peaks (Supplementary Figure 1A). Those eluted at a time similar to that reported for Pru p 7 were collected and analyzed by automated N-terminal amino acid sequencing. The peak eluting at 27.4 min produced the following sequence GSPFCDSKCGVRCSKAG, showing high sequence identity with the N-terminal region of Pru p 7. By analogy with the peach GRP, named peamaclein, the apple GRP was named applemaclein.

Applemaclein was purified to homogeneity by several chromatographic separations (Supplementary Figure 1). Its purity was assessed by a combination of three different methodologies, namely, SDS-PAGE (Supplementary Figure 2), RP-HPLC (Supplementary Figure 1D), and direct protein sequencing.

### Apple GRP Content in the Peel and Pulp of Two Different Cultivars

The GRP was purified from Annurca and Golden Delicious, peel and pulp, as described in the previous paragraph. The results obtained revealed that both the cultivars and tissues contained applemaclein. The amount of purified GRP was estimated on the basis of the molar extinction coefficient at 280 nm (6,710 M^−1^ cm^−1^). In particular, from Annurca peel and pulp, 0.21 and 0.17 mg/100 g of starting material, respectively, were purified. Lower amounts were found in the Golden Delicious peel and pulp, corresponding to 0.11 and 0.09 mg/100 g, respectively. Since the Annurca peel was found to be the richest source of this protein, applemaclein was purified from this starting material in the amounts sufficient to perform the planned studies.

### Structural Features of Apple GRP

Apple GRP was stable in gastrointestinal digestion. In fact, after incubation in SGF and SIF, the entire molecule was observed in SDS-PAGE and in RP-HPLC chromatographic profile. Supplementary Figure 3 shows the samples analyzed by SDS-PAGE. The RP-HPLC profiles of the digested protein showed a single peak eluted exactly as the untreated purified protein shown in Supplementary Figure 1D.

The similarity search performed in the Allergome database (www.allergome.org), using the experimentally obtained N-terminal protein sequence (GSPFCDSKCGVRCSKAG) showed a high sequence identity with the allergen Pru p 7 from peach and other members of the GRP family, but the absence of homologous apple proteins. A search carried out against UniProtKB (www.expasy.org) with the BLASTP algorithm on the ExPASy server, revealed 100% identity with the N-terminal region of an uncharacterized apple protein (accession number A0A498HTE5).

**Figure 1:**
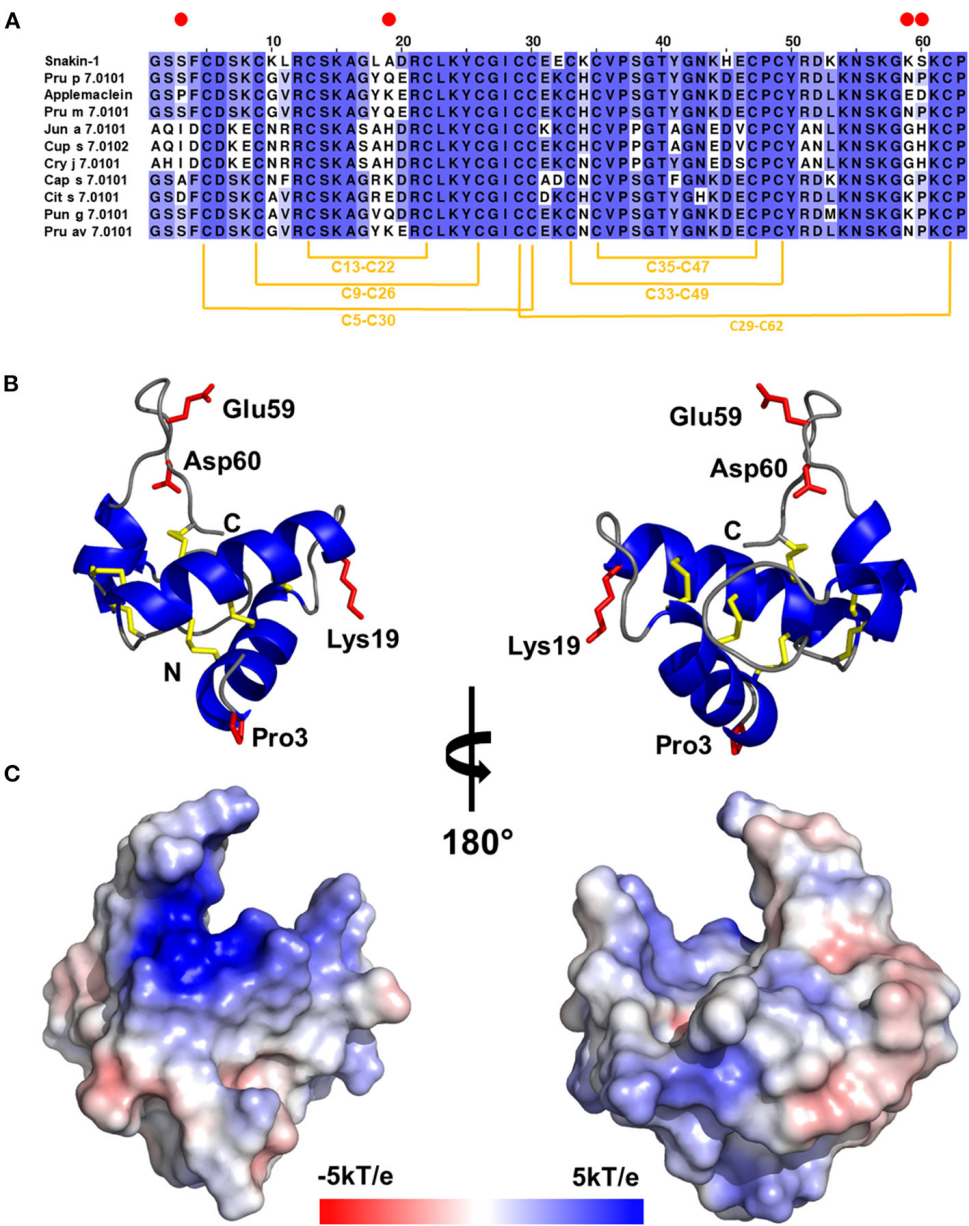
**(A)** Multiple sequence alignment of gibberellin-regulated proteins (GRPs): potato Snakin-1, peach Pru p 7.0101, applemaclein, Japanese apricot Pru m 7.0101, mountain cedar Jun a 7.0101, Mediterranean cypress Cup s 7.0102, Japanese cedar Cry j 7.0101, chili pepper Cap s 7.0101, orange Cit s 7.0101, pomegranade Pun g 7.0101, and cherry Pru av 7.0101. Red circles mark residues that are different between applemaclein and Pru p 7.0101. **(B)** Calculated model of applemaclein. Structure of the protein shown in cartoon representation. N- and C-terminal ends are labeled. The four residues that are different between applemaclein and Pru p 7.0101 are highlighted in red and shown in stick representation. **(C)** Surface representation of applemaclein with mapped charge distribution. The red color corresponds to the negatively charged areas and the blue color shows the positively charged regions.

An applemaclein is a basic protein (calculated pI = 8.5) composed of 63 amino acids, with 12 cysteine residues and a sequence-deduced molecular mass of about 7 kDa. A multiple sequence alignment of an apple GRP with homologous fruit and pollen allergenic proteins, and with the potato homolog snakin-1, is shown in Figure 1A. The alignment highlights that the highest similarity is observed when an apple GRP is compared with peach, Japanese apricot, and cherry homologous proteins with which share 94% of residue identity. This means that only four residues are substituted in the applemaclein sequence, compared with Pru p 7 (peamaclein), Pru m 7, and Pru av 7. Lower sequence identity is observed with orange and pomegranate (86%) homologs proteins. Much lower identity, ranging from 68 to 67%, was observed when apple GRP was compared with the pollen GRP, namely, Cry j 7, Cup s 7, and Jun a 7 from *C. japonica, C. sempervirens*, and *J. ashei*, respectively.

Gel filtration results indicate that the purified applemaclein is monomeric in solution, which is also consistent with the oligomeric state observed for the crystal structure of potato Snakin-1. The GRP from potato shares 81% identity with the homolog from apple. The high sequence identity between Snakin-1 and other GRPs allowed for reliable modeling of these proteins and the use of the models for the analysis of sequence conservation. However, PROFUNC search for potential ligands or DNA-binding sites did not return any significant hits. In addition, the searches for structural homologs with PDBeFOLD and DALI server have not returned any significant result except potato Snakin-1 that was used as a template for modeling. The molecular model of applemaclein is shown in Figures 1B,C.

### Direct IgE Binding to Fruit and Pollen GRP

**Figure 2:**
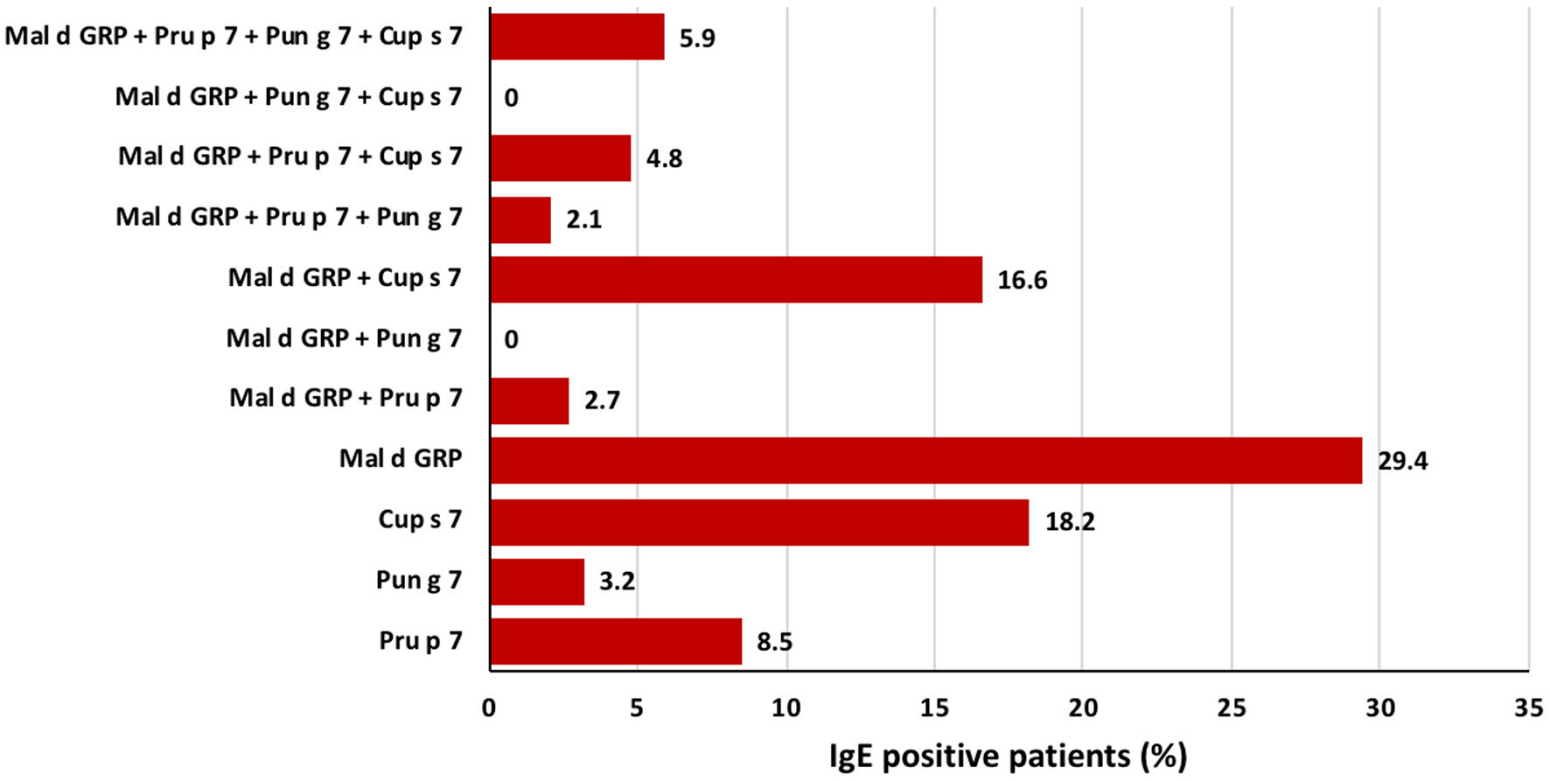
Frequency of sensitizations in a population of 187 patients of Italy IgE-positive to at least one of the four analyzed GRP, namely apple GRP, Pru p 7, Pun g 7, and Cup s 7. Starting from the bottom, the figure shows the percentage of mono-sensitized patients, then those bi-sensitized, those tri-sensitized, and the last column display those sensitized to all the four analyzed GRPs.

Out of 4,721 consecutive random patients complaining of allergic symptoms toward any food or inhalant source and tested with the FABER system, the clinical history was available for 1,000 of them (as shown in Supplementary Figure 4 for details). Total 115 (2.43%) of 4,721 patients proved to have specific IgE recognizing applemaclein. The patients IgE positive to one or more of the four GRP spotted on the FABER biochip were 187 (4.0%). Among them, 115 (61.5%) had specific IgE recognizing applemaclein, 61 (32.6%) were positive to Pru p 7, and 30 (16.0%) and 99 (52.9%) were IgE positive to Pun g 7 and Cup s 7, respectively. Further analysis highlighted that 55, 34, 16, and 6 patients were monosensitized to apple GRP, Cup s 7, Pru p 7, and Pun g 7, respectively (Figure 2, Supplementary Table 1). The clinical history was available for 37 patients sensitized to applemaclein (Table 1). Among them, one patient (n. 24, Table 1) only reported allergic reactions against apple, but the patient was also IgE positive to other allergens, such as nsLTP, therefore it was not possible to identify the culprit molecule. Similarly, seven patients stated they did not eat the apple, but they were also positive to Pru p 3. Therefore, most (29 of 37, 78.4%) of the patients sensitized to apple GRP stated they were used to eat an apple and did not report allergic reactions against this fruit, although some of them experienced symptoms after consumption of some seeds and fresh fruits. One patient (n. 32, Table 1) referred allergic reaction to peach and other plant foods. The patient was sensitized to Pru p 7 but to none of the other tested peach allergens (Pru p 3, the peach pulp extract, and the peach peel extract).

**Table 1:** Immunological and clinical data of 37 patients sensitized to the apple gibberellin-regulated protein (GRP) in Italy.

**Patient details**			**FABER 244 (FIU)** ^a^						**Offending foods and symptoms**		
	**Sex**	**Age**	**Pru p 7**	**Pun g 7**	**Apple GRP**	**Cup s 7**	**Pru p 3**	**Mal d 1**	**Apple allergy^b^**	**Offending fruit/seed^c^**	**Symptoms^d^**
1	F	40	0.92	12.86	3.19	0.92	7.24	5.49	NE	0	GI, U, R
2	F	27	0.68	0.23	0.23	0.23	2.91	0	NE	P, C, H, L	OAS, U, Ang, R
3	M	8	27.89	14.29	3.41	0	2.70	0	NE	W, A, PI	GI, Ang, R
4	M	61	0.25	0	0.25	0.25	0	0,91	0	0	R, A
5	M	39	0	0	0.47	0.47	0	0	0	0	Ang, R
6	M	48	1.78	0	1.02	1.53	0.76	0	NE	PE, T	OAS, Ana
7	F	50	2.49	0	2.47	3.71	6.19	0	NE	H	GI, R, C, A, E
8	F	27	0.19	0	0.19	0	0.74	0	NE	LE, E	OAS, U, Ang, R, C, A, E
9	F	56	0	0	0.71	0	0	0	0	0	R
10	F	55	0	0	0.39	0.58	0.97	0	0	H, S, CH, PE, PL, PIN, AP, PR, B, R, P, W, ST	U, Ang, R
11	F	31	0	0	2.82	3.37	0	0	0	0	GI, R
12	F	23	0	0	0.59	22.07	2.13	6,7	NE	O, T, W, H, PE	OAS, Ang, A
13	F	39	0	0	1.12	0.75	0	0	0	0	R, A
14	M	12	0	0	1.12	1.67	1.67	0	0	LE	R, C
15	F	73	0	0	0.63	0.94	0	0	0	0	A
16	M	57	0	0	1.44	0.24	0	0	0	0	U
17	F	65	0	0	0.76	0	0	0	0	0	R, C
18	F	28	0	0	0.71	0	0	0	0	0	GI
19	M	15	0	0	0.94	0	0	0	0	0	R
20	M	33	0	0	0.68	0	0	0	0	0	R
21	M	14	0	0	0.89	0	0	0	0	0	R, C
22	F	32	0	0	1.02	0	0	0	0	0	U, R
23	F	40	0	0	0.66	0	0	0	0	T	U, R
24	M	10	0	0	0.59	0	0.91	0	1	A, PL, OR, H, T, APL, Ch	OAS, U, Ang, E
26	M	35	0	0	10.53	0	0	0	0	0	R, C, A
27	F	32	0	0	0.56	0	0	0	0	0	U, R, C
28	F	61	0	0	0.35	0	0	0	0	0	R, C, A
29	F	13	0	0	0.5	0	0	0	0	0	R, E
30	F	5	0	0	0.64	0	0	0	0	0	R, A
31	M	14	0	0	0.11	0.2	0	0	0	0	R, C, A
32	F	34	35.47	0	9.29	23.12	0	0	0	PE, BL, W, E	GI, U, Ang, R
33	F	19	0	0	2.35	1.95	0	0	0	0	R
34	F	41	0	0	1.3	0	0	0	0	H,W	U
35	M	12	9.85	5.2	2.98	0.9	1.80	0	0	W	Ang, R, A, E
36	F	8	0	0	0.31	0.93	0	0	0	PI	U, R, A
37	F	15	0	0	0.39	0.76	0	0	0	0	U, R

a *FIU, FABER International Units, positive value FIU≥0.01*;

b *NE, not eaten*.

c *A, almond; PIN, pineapple; AP, apricot; APL, apple; B, blackberry; BL, blueberry; C, corn; CH, cherry; E, eggplant; H, hazelnuts; L, lettuce; LE, lentil; O, olive; OR, orange; P, peanut; PE, peach; PI, pistachio; PL, plum; PR, pear; R, rasberry; S, strawberry; ST, string bean; T, tomato; W, walnut*.

d *A, asthma; Ang, angioedema; C, conjunctivitis; E, eczema; GI, gastrointestinal symptoms; OAS, oral allergy syndrome; R, rhinitis; U, urticaria*.

### IgE-Binding Inhibition

**Figure 3:**
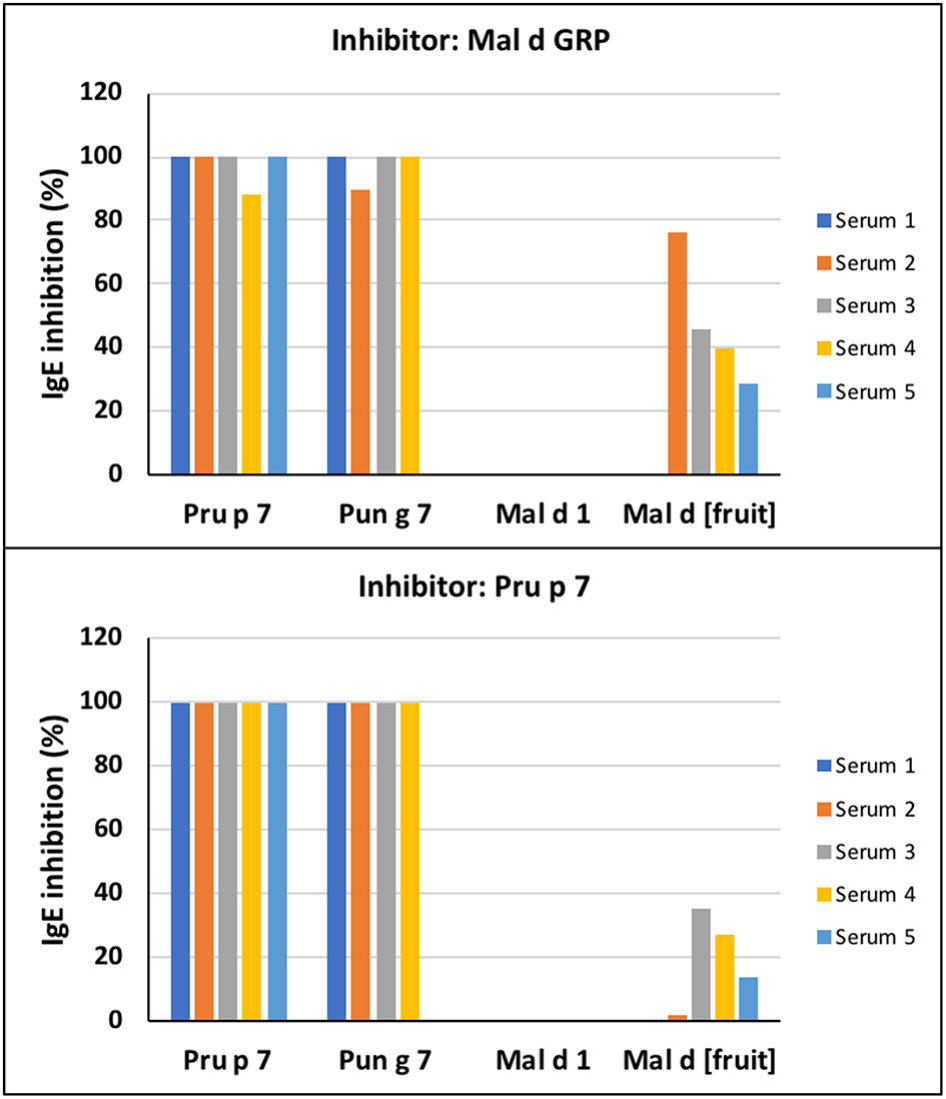
Immunoglobulin E -binding competition. The inhibition assays were performed on the FABER testing system with the SPHIAa method. Five individual sera of patients sensitized to Pru p 7 were used as providers of specific IgE (Supplementary Table 1). The analyzed antigens spotted on the biochip are Pru p 7, Pun g 7, Mal d 1, and the apple extract, Mal d [Fruit]. The inhibitors of IgE binding were applemaclein **(upper panel)** and Pru p 7 **(lower panel)**.

The SPHIAa protocol was applied to the FABER system to run specific IgE inhibition experiments using the purified applemaclein and Pru p 7. The five sera used for these experiments were from the patients sensitized to Pru p 7. Four of them were also IgE positive to Pun g 7 (Supplementary Table 2). The clinical history was available for two of these 5 patients, namely patients 2 and 5. These patients had allergic reactions to peach, or peach juice, and to a limited number of fruits and seeds. Similar to Pru p 7, an apple GRP completely, or almost completely, inhibits the IgE binding to Pru p 7 (Figure 3). In fact, the pre-incubation of these proteins with each one of the five selected sera was effective in inhibiting the IgE binding to the peach and pomegranate GRPs spotted on the FABER biochip. In addition, a partial inhibition of the apple extract was detected. As a control, the lack of IgE-binding inhibition on the apple Bet v 1-like protein, Mal d 1, is reported.

## Discussion

Applemaclein was found in quite similar amounts in the peel and pulp of both the analyzed cultivars, one (Annurca) is common in the South of Italy and the second (Golden Delicious) is a widespread commercial one. The protein shows a high sequence identity (94%) with Pru p 7, representing the prototype of allergenic GRP (2, 3, 5). Pru p 7 has mainly helical structure in the N-terminal region and the C-terminal appears flexible in solution by NMR analysis (1). The detected structural features suggest a possible shift of the molecule to a significantly more structured form following the interaction with a ligand, likely negatively charged surfaces, such as biological lipids/membranes (1). The two N-terminal helical stretches encompass residues 4–16 and 25–30. Compared with Pru p 7, the first amino acid substitution (residue 3) in an apple GRP is just before the first helical strech, the second substitution (residue 19) is placed between the first and the second helical stretch, whereas residues 59–60 are placed in the C-terminal loop region (Figures 1A,B). Therefore, the structural differences between the peach and apple GRP rely on these four residues that could be directly, or indirectly, involved in IgE-binding individual properties. Based on our modeling results, we suggest that most likely residues 59 and 60 are the most important in relation to the differences in IgE binding between applemaclein and Pru p 7. Our prediction is based on the fact that the neutral residues Asn59 and Pro60 of Pru p 7 are replaced with the two negatively charged residues Glu59 nd Asp60, respectively (Figure 1). Therefore, the surface properties of the corresponding C-terminal loop regions differ significantly. Additionaly, it was shown that the C-terminal loop region displays the highest mobility (27), therefore it is likely that a drastic change in the amino acid composition will also impact the conformation of this region and as a result, alter a surface region that potentially is recognized by IgE.

An apple GRP was recognized by specific IgE of the allergic subjects and efficiently competed with the peach and pomegranate GRP for IgE binding, producing a complete inhibition on both the homologous proteins. In a random and heterogeneous population of 4,721 Italian suspected allergics, the frequency of sensitization to the apple GRP was higher than that observed for the peach and pomegranate, but much more similar to that of Mediterranean cypress homologous protein. Nevertheless, in a population of 37 sensitized patients of Italy for which a clinical history was available, only one patient reported allergic reactions to apple, whereas some others did not report symptoms, but excluded this fruit from their diet. It is worth noting that the patient reporting allergic reactions, as well all the patients refusing the apple consumption, were not monosensitized to the GRP, but they were also IgE positive to nsLTP and/or Mal d 1. Therefore, it was not possible to associate the exclusion of apples from their diet to GRP and/or nsLTP and/or to other allergens. In addition, most of the 37 patients of Italy IgE positive to applemaclein, some of them at least apparently monosensitized to GRP, reported the consumption of apple without allergic symptoms connected to this fruit. In contrast, a Japanese study described the detection of patients monosenstized to GRP reporting allergic reactions against several fruits, frequently, such as apple (4), although peach was the fruit most frequently (92.3%) inducing allergic symptoms. Unfortunately, due to the insufficient amount of available sera, we could not investigate the possibility that the high levels of IgG/IgG4 blocking antibodies affected the results obtained in the analyzed population of Italy. Nevertheless, the mismatch between positive sensitization and the lack of reported clinical symptoms to apple claims further future investigations. Furthermore, in addition to the GRP allergens, it will be interesting to perform similar studies on other plant allergens and make a comparative analysis.

The researchers from France described the peach GRP as a major allergen in the patients allergic to peach from southern France, where the exposure to cypress pollen is high, with a prevalence of Pru p 7 sensitization exceeding 60% (9, 28). In contrast, a recent study reported that the allergy and sensitization to foods secondary to cypress pollen allergy appeared as rare phenomenon in the Italian population and that most of such patients react to peach, rather than other *Rosaceae* fruits (29). Therefore, discrepant results concerning the allergenic properties of GRP can sometimes be found in the literature. It seems that the same protein displays a different behavior, becoming a major or minor allergen, which may cause mainly localized or systemic allergic reactions, depending on the population/geographical area considered.

It is therefore conceivable that some factors, or a combination of them, can influence the GRP behavior and the response of our immune system. A great relevance has been so far given to the primary structure of allergenic proteins and certainly, it is of utmost importance and can explain the co-recognition and cross-reaction occurrence (30, 31). However, an increasing collection of data suggests that the final result, in the terms of sensitization and allergic reactions, could be affected also by other factors. For instance, a study performed in a Japanese population revealed that in more than half of the patients monosensitized to GRP (61.5%), exercise or aspirin intake acted as cofactors enhancing the allergic reaction onset (4). The influence of these factors, and also of additional ones, including alcohol, non-steroidal anti-inflammatory drugs, estrogens, antiacids, temperature, and compounds from *Cannabis sativa*, was reported for some allergens (32), especially for nsLTP (33, 34). nsLTP was described as the most frequent sensitizer in the Italian subjects with food-dependent exercise-induced anaphylaxis (FDEAIA) (35). It was reported that the allergenicity of nsLTP, as well as that of other allergens with the function of hydrophobic molecule carrier, could be influenced by the bound ligand which may act as an adjuvant and drives the immune system toward a type 2 (pro-allergenic) response (36, 37). For instance, the interaction of Pru p 3 with selected fatty acids was described to increase its IgE binding capacity, whereas the binding of some other lipids did not have the same effect (38). Different hydrophobic molecules have been suggested to bind nsLTP from several sources (39, 40). In fact, the nsLTP fold enables the formation of a hydrophobic tunnel-like cavity where hydrophobic ligands with different sizes can be accommodated (31, 37).

Similar to nsLTP, a GRP structure is predicted to form a cavity (12) where a ligand, such as biological lipids/membranes, can be bound (1). This is in agreement with the applemaclein modeling results (Figure 1C) showing that the surface of applemaclein is very irregular and there are some grooves/clefts that may potentially accommodate some small molecular ligands. However, we were not able to indentify such ligands. Due to its positive charge, the GRPs may interact with some negatively charged molecules, and therefore it is not surprising that they are associated with the membranes (41). It was also demonstrated that the GRPs have antimicrobial properties and are able to inhibit the growth of various bacterial and fungal pathogens (42, 43). In addition, the GRPs may be involved in the interactions with foreign proteins, such as bacterial adhesion protein from *Pseudomaonas fluorescense* (44), or interact with plant sucrose transporter SUT-1 (45). Generally, these proteins are implicated in many biological functions in plants, however, the molecular mechanism of their functions is still not understood. While the automated searches have not provided any clear hint on applemaclein molecular function, the available literature consistently reports that the GRPs are not susceptible to digestion and are quite stable (1). In line with the literature, applemaclein also proved to be resistant to trypsin and pepsin digestion. The combination of this information with the structural features of applemaclein (several disulfide bridges, small size and a presence of a loop that can protrude to an active site of an enzyme) hint that the protein may potentially act as a protease inhibitor or an enzyme inhibitor. For example, it was shown that Snakin-Z isolated from *Zizphus jujuba* fruits is able to inhibit human acetylcholinesterase and butyrylcholinesterease (46). The model of applemaclein (Figure 1B) suggests that one of the lysine residues (Lys57) is located on a C-terminal loop region of the protein and that the localization of this residue is somewhat similar to the position of a lysine residue present in bovine trypsin inhibitor, which blocks the active site of human trypsin (47). However, while there are some structural similarities between the applemaclein and small protein protease inhibitors, our experimental results have not confirmed the inhibition of bovine trypsin by the apple GRP, nor the formation of a stable complex between these two proteins.

In analogy with nsLTPs, which are described as the most common triggers and responsible for the main allergies in the Mediterranean area, we can speculate that the GRPs could represent important allergens frequently causing severe symptoms especially in the areas, such as Japan, but they could be less important in other geographical areas, such as Italy. Further studies are needed to identify the ligand(s) of applemaclein, and of GRPs in general, and to understand whether the bound molecules play a role in the modulation of IgE binding and the triggering of allergic reactions.

## Data Availability Statement

Additional data are available in Supplementary Material. Further inquiries can be directed to the corresponding author.

## Ethics Statement

Ethical review and approval was not required for the study on human participants in accordance with the local legislation and institutional requirements. Written informed consent to participate in this study was provided by the participants' legal guardian/next of kin.

## Author Contributions

LT and RA: methodology and writing. CA and MT: data analysis and writing. IG: methodology. MC: conceptualization, funding acquisition, data analysis, and writing. AM: patient selection, data analysis, and funding. MAC: conceptualization, funding acquisition, data analysis, and writing. All authors have read and agreed to the published version of the manuscript.

## Funding

This study was partially supported by ADL s.r.l., on the basis of an Agreement of Scientific Collaboration. MC was partially supported by the R01AI077653 grant from the National Institute of Allergy and Infectious Diseases. The content is solely the responsibility of the authors and does not necessarily represent the official views of ADL and of the National Institutes of Health.

## Conflict of Interest

IG and LT were employees of ADL s.r.l. when this study started, currently they are employees of IBBR-CNR. MT and MAC receive funding from ADL s.r.l. The remaining authors declare that the research was conducted in the absence of any commercial or financial relationships that could be construed as a potential conflict of interest.

## Publisher's Note

All claims expressed in this article are solely those of the authors and do not necessarily represent those of their affiliated organizations, or those of the publisher, the editors and the reviewers. Any product that may be evaluated in this article, or claim that may be made by its manufacturer, is not guaranteed or endorsed by the publisher.
