# Supplementary material for: When the Frequencies of Sensitization and Elicitation of Allergic Reaction Do Not Correlate—The Case of Apple Gibberellin-Regulated Protein Tested in an Italian Population
Source: Front Allergy. 2021 Oct 21;2:745825. doi: 10.3389/falgy.2021.745825 (PMC8974745; doi:10.3389/falgy.2021.745825)
Supplement: Supplementary file 1 [file Data_Sheet_1.docx]

Supplementary Material

**When the frequencies of sensitization and elicitation of allergic reaction do not correlate - the case of apple gibberellin-regulated protein tested in an Italian population**

**Lisa Tuppo^1,2^, Claudia Alessandri^2,3^, Ivana Giangrieco^1,2^, Maurizio Tamburrini^1^, Ricardo Hernandez Arriaza^4^, Maksymilian Chruszcz^4^, Adriano Mari^2,3^, Maria Antonietta Ciardiello^1*^**

^1^Institute of Biosciences and BioResources (IBBR), CNR, I-80131 Naples, Italy

^2^Allergy Data Laboratories (ADL) S.r.l., Latina, Italy

^3^Associated Centers for Molecular Allergology (CAAM), Rome, Italy

^4^Department of Chemistry and Biochemistry, University of South Carolina, Columbia, SC 29208, USA;

*** Correspondence:**

Maria Antonietta Ciardiello,

Tel: +39 081 6132573

Fax: +39 081 6132646

Email: [mariaantonietta.ciardiello@ibbr.cnr.it](mailto:mariaantonietta.ciardiello@ibbr.cnr.it)

**Running Title:**

Apple GRP

**Keywords:** apple GRP, applemaclein, food allergy, Pru p 7, IgE-binding, sensitization frequency, molecular model





**Supplementary Figure 1.** Purification of applemaclein. (A) RP-HPLC analysis of the peel extract. Black arrow indicates the peak identified as applemaclein. (B) Separation of the extract by cation exchange chromatography and (C) gel filtration. Black bar in B and C indicates the fractions containing applemaclein. (D) RP-HPLC analysis of the purified protein (30 μg).





**Supplimentary Figure 2.** SDS-PAGE of 25 μg of apple peel extract (A) and 5 μg of purified applemaclein. M, molecular mass markers.

**Apple GRP stability to the simulated gastric and intestinal digestion**

*In-vitro* gastric digestion of the purified protein was performed as described by Moreno et al. (1). Apple GRP (30 μg) was solubilized in simulated gastric fluid (SGF) (0.15 M NaCl adjusted to pH 2 with 1 M HCl) before the addition of pepsin (porcine pepsin; Roche Diagnostics GmbH, Mannheim, Germany) at the enzyme/substrate ratio of 1:20 (w/w). The digestion was performed at 37°C. Aliquots were taken at 0 and 120 min and digestion was stopped by raising the pH to 7.4 by addition of 50 mM Na-phosphate buffer pH 7.4. Samples were then loaded onto a Vydac (Deerfield, IL, USA) C8 column for RP–HPLC analysis using a Beckman System Gold apparatus and onto 15% reducing SDS-PAGE (**Supplementary Figure 3A**). As a control, 30 μg of rPhl p 7 (Indoor Biotechnologies, Cardiff, UK) was simultaneously digested following the same procedure and results are shown in **Supplementary Figure 3B**).

For intestinal digestion, porcine trypsin and bovine chymotrypsin (Roche Diagnostics GmbH) were used. Simulated intestinal fluid (SIF) was prepared as described in the United States Pharmacopeia (2), and consists of a mixture of trypsin and chymotrypsin in 0.05 M potassium-phosphate buffer pH 6.8. Digestions were performed at 37°C, at an enzyme/substrate ratio of 1:50 (w/w), both for trypsin and chymotrypsin. Aliquots of the digested samples were withdrawn at 0 and 120 min and analyzed by RP–HPLC and by 15% reducing SDS-PAGE (**Supplementary Figure 3A**). As a control, rPhl p 7 (Indoor Biotechnologies) was simultaneously digested following the same procedure and results are shown in **Supplementary Figure 3B**).


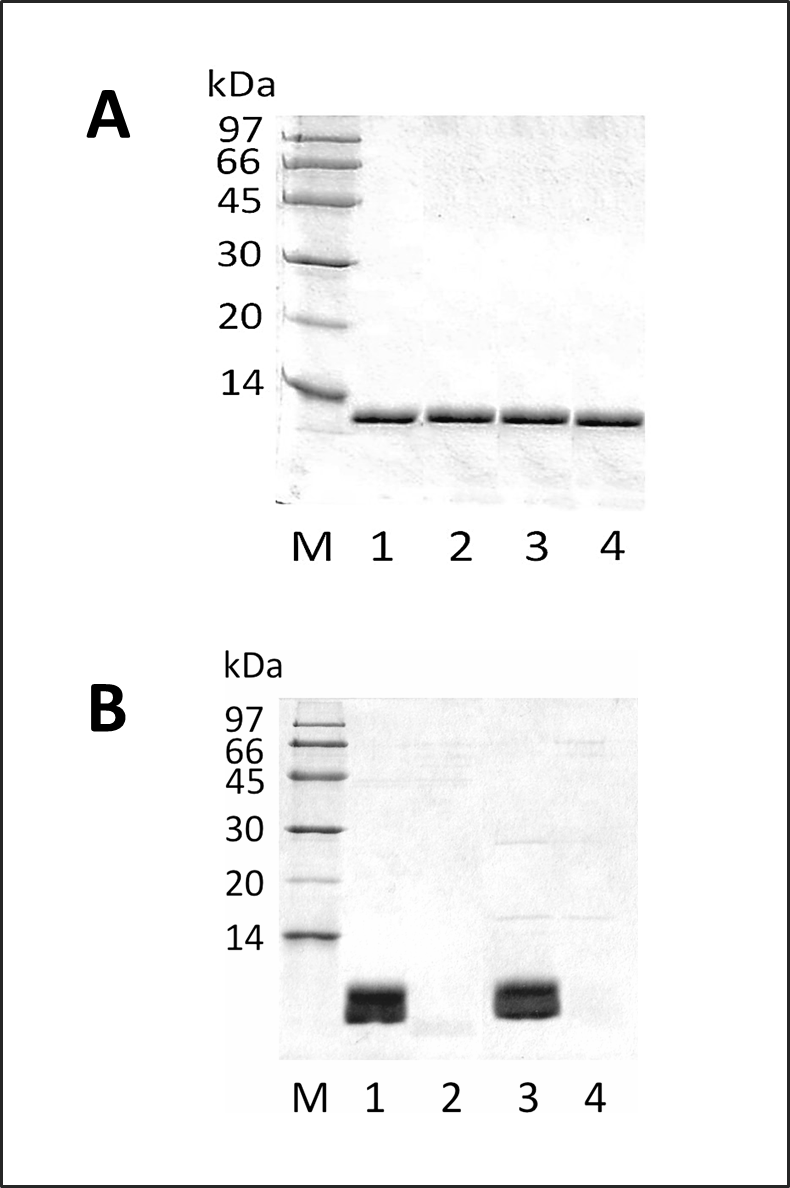


**Supplementary Figure 3**. A, SDS-PAGE of apple GRP treated with SGF and SIF. M, molecular mass markers; 1, zero min in SGF; 2, 120 min in SGF; 3, zero min in SIF; 4, 120 min in SIF.

B, SDS-PAGE of rPhl p 7 treated with SGF and SIF. M, molecular mass markers; 1, zero min in SGF; 2, 120 min in SGF; 3, zero min in SIF; 4, 120 min in SIF.


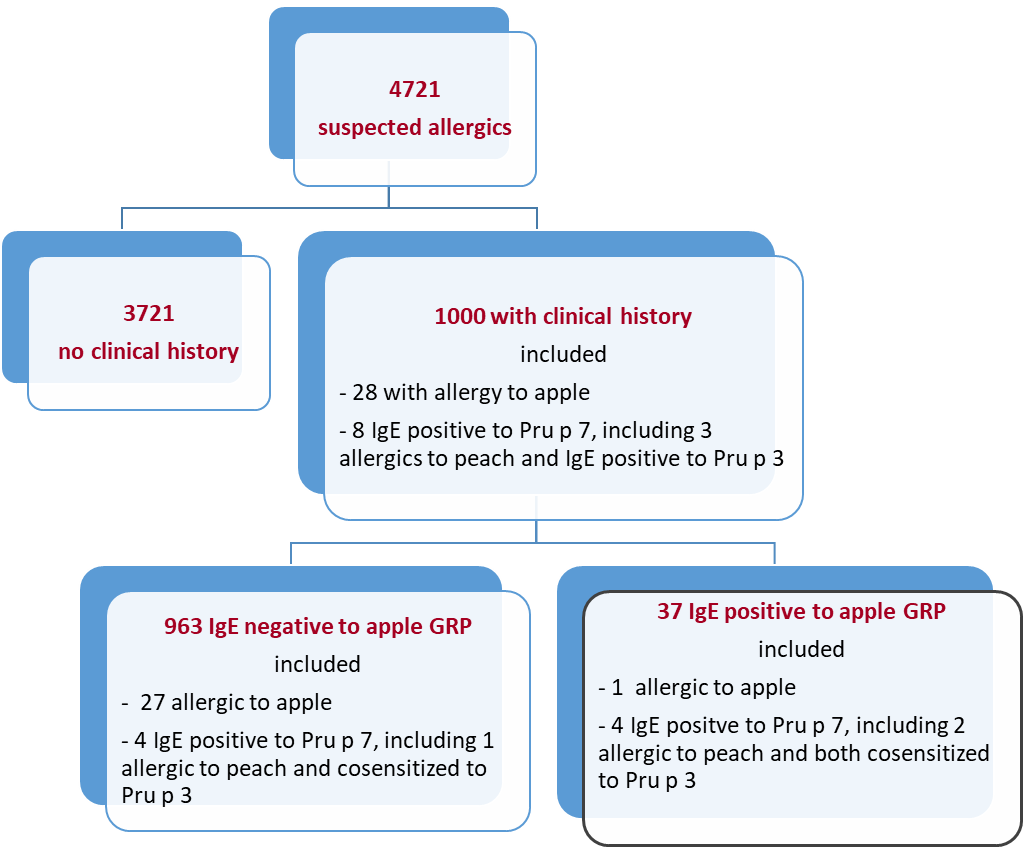


**Supplementary Figure 4.** Flow chart showing some features of the Italian population analyzed in this study.

**Supplementary Table 1**. Sensitization prevalence in a population of 187 Italian patients IgE-positive to at least one of the four analyzed GRP.

| **Number of sensitizations** | **Apple-GRP** | **Pru p 7** | **Pun g 7** | **Cup s 7** | **Patients** | **Prevalence (%)** |
| --- | --- | --- | --- | --- | --- | --- |
| 1 | + | - | - | - | 55 | 29 |
|  | - | + | - | - | 16 | 9 |
|  | - | - | + | - | 6 | 3 |
|  | - | - | - | + | 34 | 18 |
| 2 | + | + | - | - | 5 | 3 |
|  | + | - | + | - | 0 | 0 |
|  | + | - | - | + | 31 | 17 |
|  | - | + | + | - | 2 | 1 |
|  | - | + | - | + | 7 | 4 |
|  | - | - | + | + | 0 | 0 |
| 3 | + | + | + | - | 4 | 2 |
|  | + | + | - | + | 9 | 5 |
|  | + | - | + | + | 0 | 0 |
|  | - | + | + | + | 7 | 4 |
| 4 | + | + | + | + | 11 | 6 |
|  |  |  |  |  | 187 | 100 |

**Supplementary Table 2.** Features of patients and sera used in IgE-inhibition experiments. The clinical history was available for patients 2 and 5 only.

| **PATIENT DETAILS^a^** | | | | | **FABER 244 (FIU)^b^** | |
| --- | --- | --- | --- | --- | --- | --- |
| **Number** | **Gender** | **Age** | **Symptoms** | **Offending food** | **Pru p 7** | **Pun g 7** |
| **1** | M | 16 | NA | NA | 5.04 | 10.80 |
| **2** | F | 8 | R, U, OAS, AP | nuts, kiwifruit, peach juice | 7.42 | 8.05 |
| **3** | F | 35 | NA | NA | 12.94 | 16.62 |
| **4** | F | 15 | NA | NA | 28.45 | 4.42 |
| **5** | F | 28 | R, U,ANG, OAS, G | peach, nuts, melon, kiwifruit, corn, tomato | 31.26 | 0 |

^a^Patient 2 eats fresh fruits with and without peel, except kiwifruit. Patient 5 tolerates apple.

NA, not available; Ang, angioedema; AP, abdominal pain; G, gastritis; OAS, oral allergic syndrome; R, rhinitis; U, urticaria.

^b^FABER International Units; positive value FIU ≥ 0.01

**REFERENCES**

1. Moreno FJ, Mellon FA, Wickham MS, Bottrill AR, Mills EN. Stability of the major allergen Brazil nut 2S albumin (Ber e 1) to physiologically relevant in vitro gastrointestinal digestion. FEBS J. 2005; 272:341–52.

2. Stippler E, Kopp S, Dressman JB. Comparison of US pharmacopeia simulated intestinal fluid TS (without pancreatin) and phosphate standard buffer pH 6.8, TS of the international pharmacopoeia with respect to their use in in vitro dissolution testing. Dissolut Technol 2004; 6–10.
